# The infectious thyroid nodule: a case report of mucormycosis associated with ibrutinib therapy

**DOI:** 10.1186/s40463-019-0376-1

**Published:** 2019-10-16

**Authors:** Marco A. Mascarella, Lorne Schweitzer, Mahmoud Alreefi, Jennifer Silver, Derin Caglar, Vivian G. Loo, Keith Richardson, Philippe Dufresne, Todd C. Lee, Nader Sadeghi

**Affiliations:** 1 Department of Otolaryngology-Head and Neck Surgery, McGill University Royal Victoria Hospital, McGill University Health Centre, Glen Site, 1001 Boul. Decarie, D05.5704, Montreal, QC H4A 3 J1 Canada; 2 Department of Epidemiology, Biostatistics and Occupational Health, McGill University, Montreal, Canada; 3 Division of Infectious Diseases, Department of Medicine, McGill University Health Centre, Montreal, Canada; 4 Department of Pathology, McGill University Health Centre, Montreal, Canada; 5 Laboratoire de Santé Publique du Québec, Sainte-Anne-de-Bellevue, Canada

**Keywords:** Mucormycosis, Thyroid nodule, Ibrutinib, Acute invasive fungal infection

## Abstract

**Background:**

Acute invasive fungal infections of the head and neck secondary to tyrosine kinase inhibitors are rare and potentially life-threatening events.

**Case presentation:**

We report a case of mucormycosis of the thyroid gland in a patient known for chronic lymphocytic leukemia receiving ibrutinib who presented with a rapidly growing thyroid nodule and dysphonia. An acute invasive fungal infection was identified on a core needle biopsy; mucormycosis was confirmed on culture. The patient was successfully treated with surgical debridement and long-term antifungal therapy.

**Conclusion:**

Patients on ibrutinib may be at risk of acute invasive fungal infections of the head and neck.

## Introduction

Invasive mucormycosis is an infection affecting the immunocompromised population for which the mainstay of therapy is surgical debridement and antifungal therapy. Despite early and aggressive therapy, it continues to have a high mortality rate. While an association between treatment with ibrutinib and invasive fungal infections has been described, there have been few case reports of its association with mucormycosis and none affecting the head and neck region [1–3]. In this case report, we describe a patient receiving ibrutinib treatment for chronic lymphocytic leukemia (CLL) who presented with a rapidly enlarging thyroid nodule, dysphagia, and dysphonia and was found to have thyroid mucormycosis requiring surgical debridement and systemic antifungal treatment.

## Case report

**Fig 1:**
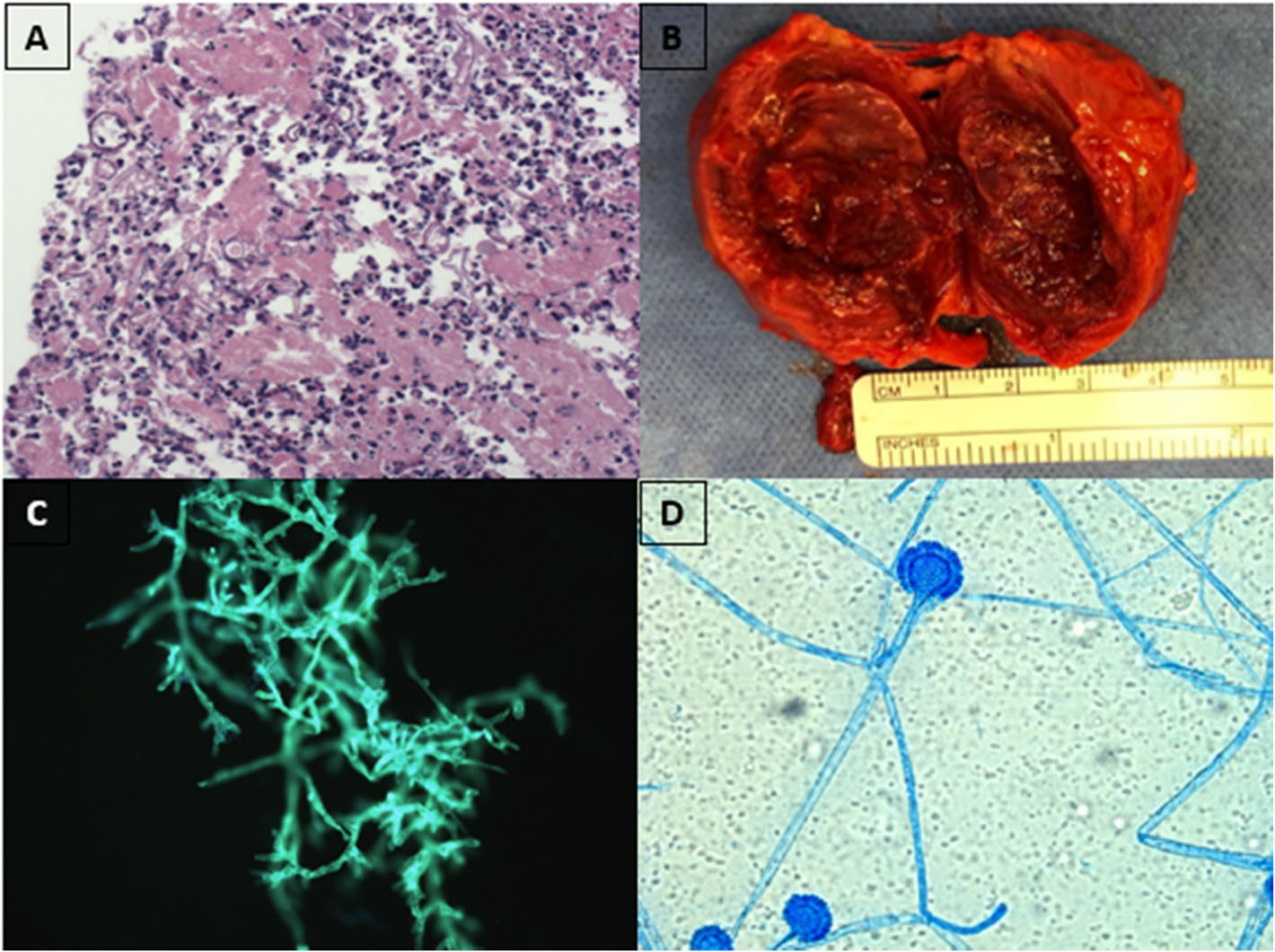
Clinical, pathologic and microbiologic evaluation of thyroid mucormycosis. **a**: Histopathology of specimen showing fungal hyphae with wide-angle branching in background of acute inflammation with giant cell body reaction. **b**: Postoperative specimen showing partially encapsulated lesion with necrotic center. **c**: direct calcuoflor-white staining from specimen showing pauciseptate hyphae branching at 90-degree angles. **d**: direct examination of culture specimen of *Cunninghamella* spp. after 48 hours of incubation on Sabouraud agar.

We report the case of a 79-year-old male who was referred to a head and neck surgeon for a rapidly progressive midline neck mass and dysphagia. His past medical history was significant for high-risk CLL for which he had begun first-line therapy with ibrutinib 2 months prior for progressive adenopathy and constitutional symptoms. On examination, the patient had a 3 cm midline neck mass, which was fixed to the larynx with palpable cervical lymphadenopathy and an immobile left vocal cord. The patient was initially admitted to an outside hospital and received empiric intravenous piperacillin-tazobactam and vancomycin for 2 weeks without any improvement. Given the ongoing and rapid progression coupled with failure of antibiotic therapy, the lesion was considered to be suspicious for an aggressive thyroid cancer. Neck ultrasonography revealed a left thyroid lobe completely replaced by a solid nodule of 5.0 × 4.4 × 3.7 cm with a central cystic component with ipsilateral cervical lymphadenopathy. An ultrasound guided fine needle aspiration was performed and found to be suspicious for poorly differentiated thyroid cancer. Axial imaging of the neck showed a large necrotic thyroid mass invading the sternocleidomastoid, cricoid cartilage and possibly skin, with extensive bilateral adenopathy. A core needle biopsy revealed necrotic tissue with adjacent acute inflammation, histocytic giant cell reaction and ribbon-shaped hyphae on hematoxylin and eosin staining consistent with *Mucorales* spp. (Fig. 1a). The patient was urgently taken for surgical debridement. During debridement, the cricoid cartilage was invaded with a 1 cm intralumenal opening requiring sternocleidomastoid flap repair (Fig. 1b). The wound was packed and left open for re-exploration in the intensive care unit, which revealed healthy granulation tissue. Systemic imaging did not reveal any other foci of disease. He was extubated on the seventh post-operative day.

A sample of the excised thyroid tissue was received fresh in the microbiology laboratory, processed without grinding, plated on Sabouraud agar and blood agar and incubated at 28 °C. A direct calcofluor blue stain revealed branching, pauci-septate hyphae at ninety-degree angles (Fig. 1c). It initially failed to grow and was replated from remaining tissue, which resulted in growth after 48 h. Microscopic examination revealed pauci-septate hyphae with microconidia in rosettes visually consistent with *Cunninghamella* spp. (see Fig. 1d). This was definitively identified as *Cunninghamella bertholletiae* by 18S ribosomal sequencing. Resistance testing revealed sensitivity to posaconazole, amphotericin B and itraconazole, with resistance to voriconazole, caspofungin, micafungin, anidulafungin, 5-fluorocytosine and fluconazole. The patient was initially treated with intravenous liposomal amphotericin B at a dose of 5 mg/kg; however, this had to be discontinued after 2 weeks of therapy due to acute kidney injury. He was subsequently treated with posaconazole, as he could not swallow extended-release tablets due to dysphagia, for a further 12 weeks. He remained disease-free 8 months after discontinuing antifungal therapy without significant symptoms apart from a vocal cord paralysis.

On further history, it was determined that a large pile of mulch was present on the patient’s property since the previous fall, however he had not participated in any gardening activities. While this is thought to be the source of exposure to *Cunninghamella bertholletiae*, we were unable to isolate it from a provided sample of mulch.

## Discussion and conclusion

Fungal thyroiditis due to *Mucorales* spp. is exceedingly rare with only 11 previous cases reported in the literature. Only two previously reported cases, a renal transplant patient in India and a child undergoing treatment for acute lymphoblastic leukemia in Poland, involved isolated thyroid disease [4, 5]. The others had thyroid involvement in the context of disseminated infection [6]. Ours is the first case involving ibrutinib with the reported other cases involving solid organ transplant (*n* = 5), myeloablative therapy for acute leukemia (*n* = 6), or advanced HIV infection (*n* = 1). Of these, three were definitively identified as *Cunninghamella bertholletiae*.

While the patient did not present with traditional risk factors for mucormycosis, emerging evidence supports an association between ibrutinib therapy and invasive fungal infections [1, 7]. Several case reports and series have previously associated it with invasive pulmonary Aspergillosis, cerebral Aspergillosis, *Pneumocystis jiroveci* pneumonia, and invasive cryptococcal disease. There was one case of mucormycosis reported in a retrospective case series of infections during ibrutinib therapy spanning the years 2013–2017; however, further details were not provided [1]. As in our patient, the published cases involve a particular susceptibility to infection at the initiation of therapy. Both in vitro and in vivo evidence suggest that this susceptibility is a direct result of pharmacologic inhibition of Bruton’s tyrosine kinase (BTK) by ibrutinib, however it remains unexplained why this finding has not been reported in children with X-linked agammaglobulinemia [8, 9]. This remains an area of active investigation.

The route of localization to the thyroid remains unclear; we hypothesize that the hyphae spread either through hematogenous dissemination or direct extension of inhaled spores through the trachea after exposure to aerosolized spores from the mulch in his garden. Spreading of mulch and gardening are well known risk factors for invasive mold infections and these activities are discouraged for patients at risk for prolonged neutropenia by Infectious Diseases Society of America guidelines [10]. Although we were unable to isolate a *Mucorales* spp. from a provided mulch sample and the patient did not report partaking in any gardening activities, the association between ibrutinib therapy and invasive fungal infections taken in the context of this case makes it prudent to advise patients not to partake in gardening or spreading mulch while on this therapy.

From a surgical viewpoint, a rapidly growing midline neck mass with vocal cord involvement is suggestive of aggressive thyroid malignancy. The rarity of invasive fungal infections of the thyroid likely led to delayed diagnosis in this case. Core needle biopsy is used to confirm the diagnosis of anaplastic thyroid cancer as management and prognosis differ greatly from well differentiated cancers, but in this case established the diagnosis of fungal thyroiditis. The rapidly expanding, necrotizing, locally invasive mass, which was encountered at the time of surgery, is consistent with this diagnosis. Once the diagnosis of mucormycosis was made, upfront surgical debridement of devitalized tissue with serial exploration of neck tissues became imperative to maintain disease control with the minimal amount of morbidity.

Finally, as in this case, *Mucorales* spp. might fail to grow on initial plating due to inadvertently disturbing the hyphae. All samples containing suspected *Mucorales* spp. must be plated without grinding the sample. Any sample in which fungal hyphae are seen on direct staining, but which subsequently fails to grow in the microbiology laboratory should raise the suspicion of *Mucorales* spp. for both clinicians and microbiologists and be processed accordingly.

In conclusion, we report the case of a patient with mucormycosis of the thyroid gland who presented with an enlarging thyroid nodule and dysphonia. Patients on ibrutinib may be at risk of invasive acute fungal infections of the head and neck.

## Data Availability

The data supporting the conclusion of this article can be made available upon request.

## Abbreviations

CLL: Chronic lymphocytic leukemia
